# Regulatory T Cells in Autoimmune and Viral Chronic Hepatitis

**DOI:** 10.1155/2015/479703

**Published:** 2015-05-28

**Authors:** Pascal Lapierre, Alain Lamarre

**Affiliations:** Laboratoire d'Immunovirologie, Institut National de la Recherche Scientifique, INRS-Institut Armand-Frappier, Laval, QC, Canada H7V 1B7

## Abstract

In both autoimmune liver disease and chronic viral hepatitis, the injury results from an immune-mediated cytotoxic T cell response to liver cells. As such, it is not surprising that CD4^+^ regulatory T cells, a key regulatory population of T cells able to curb immune responses, could be involved in both autoimmune hepatitis and chronic viral hepatitis. The liver can induce the conversion of naïve CD4^+^ T cells to CD4^+^ regulatory T cells and induce tolerance to locally expressed antigens. This tolerance mechanism is carefully regulated in physiological conditions but any imbalance could be pathological. An overly tolerant immune response can lead to chronic infections while an overreactive and unbridled immune response can lead to autoimmune hepatitis. With the recent advent of monoclonal antibodies able to target regulatory T cells (daclizumab) and improve immune responses and several ongoing clinical trials analysing the impact of regulatory T cell infusion on autoimmune liver disease or liver transplant tolerance, modulation of immunological tolerance through CD4^+^ regulatory T cells could be a key element of future immunotherapies for several liver diseases allowing restoring the balance between proper immune responses and tolerance.

## 1. Introduction

Chronic hepatitis can result from persistent infections with hepatotropic viruses (HBV and HCV), autoimmune responses to the liver (autoimmune hepatitis), or drug usage. While drug-induced hepatitis can generally be resolved upon drug usage cessation, autoimmune and viral hepatitis can be a lifelong illness. These can lead to fibrosis, cirrhosis, and hepatocellular carcinoma (HCC). Although autoimmune liver diseases and chronic viral infections seem diametrically opposed, both diseases result from the immune system cytotoxic response to hepatocytes (HCV and HBV being poorly cytopathic). Therefore, both conditions result from an inability to properly regulate immune responses to liver cells.

Positioned between the splanchnic and systemic venous circulations, the liver is exposed to both food-derived antigens and potential pathogens and is required to either generate effective immune responses or induce tolerance. Several observations suggest that the liver is prone to tolerance induction. For example, liver grafts can be accepted without immunosuppression in several mammals [1] and oral tolerance is abrogated when intestinal venous drainage through the liver is surgically bypassed [2]. The liver also has the unique ability amongst solid organs to directly activate naïve antigen-specific CD8^+^ T cells, an activation that can lead to Bim-dependant apoptosis through a lack of survival signal [3]. This process, leading to CD8^+^ T cell deletion, can induce T cell tolerance to locally expressed antigens [3].

One of the major mechanisms responsible for the regulation of immune responses and immune homeostasis is peripheral tolerance induction through the action of CD4^+^ regulatory T cells (Tregs) [4]. Tregs are critical to maintain immunological tolerance against self-antigens and Treg deficiency can lead to the development of autoimmune diseases [5]. While these cells are mainly known for their ability to maintain tolerance against self-antigens they have been found to regulate immune responses to pathogens, including Friend leukemia virus, HCV, HIV, and cancer [6, 7]. Tregs are produced in the thymus as a mature subpopulation of T cells but can also be induced from naive T cells in the periphery. The liver can induce the conversion of naïve CD4^+^ T cells into CD4^+^ Tregs and induce tolerance against specific antigens [8–10]. This tolerance is not restricted to liver diseases but extends systemically [8–10]. Peripheral tolerance is carefully regulated in physiological conditions but any imbalance can lead to autoimmunity or persistence of infection.

*Autoimmune Hepatitis and Regulatory T Cells*. Autoimmune hepatitis (AIH) is an organ-specific autoimmune disease of unknown aetiology that leads to an immune-mediated destruction of the hepatic parenchyma [11–13]. Despite treatment, progression to cirrhosis and end-stage liver disease occurs in 10 to 20% of cases and liver transplantation may be necessary [12, 13]. Susceptibility to autoimmune hepatitis results from the interaction of several factors including age, sex, genetic background, and environment [14–18]. AIH is more frequent in females, as in many autoimmune diseases, and follows a chronic but fluctuating course [11–13, 19]. Frequently, other autoimmune disorders are found in AIH patients and first-degree relatives [15–18].

Two subgroups of AIH have been described according to circulating autoantibodies found in these patients [11–13, 15–18]. Type 1 AIH is characterized by the presence of anti-Smooth Muscle Antibodies (ASMA) and anti-Nuclear Antibodies (ANA) [12, 15–17]. Type 2 AIH is defined by the detection of Liver-Kidney Microsomal antibody type 1 (LKM1) and/or Liver cytosol type 1 (LC1) autoantibodies [12, 15–17, 20–22]. LKM1 and LC1 autoantibodies target, respectively, liver-expressed cytochrome P450 2D6 (CYP2D6) and formiminotransferase-cyclodeaminase (FTCD) [22–25]. While B cell autoantigens targeted in type 1 AIH are not liver-specific, CYP2D6 and FTCD, targeted in type 2 AIH, are mostly expressed by hepatocytes [22–25]. In type 2 AIH patients, circulating CD4^+^ T cells specific to CYP2D6 have been found and an overlap of B and CD4^+^ T cell epitopes has been described [26]. CD8^+^ T cells specific to CYP2D6 have also been reported and target multiple epitopes that overlap B cell epitopes [27]. Interferon-gamma secretion by these CYP2D6-specific CD8^+^ T cells correlates with biochemical and histological markers of disease activity [27]. These results suggest that the anti-CYP2D6 immune response is intricately linked to the pathogenesis of type 2 AIH. The T cell response to FTCD, the other targeted autoantigen in type 2 AIH patients, remains to be studied.

Molecular mimicry between liver autoantigens and viral proteins has been frequently proposed as a possible initiating mechanism for autoimmune hepatitis but has never been confirmed [23, 25]. Several murine experimental models of autoimmune hepatitis were developed based on the hypothesis that molecular mimicry could induce a break of tolerance against liver autoantigens and lead to the development of autoimmune responses. However, this hypothesis assumes that the targeted autoantigen is mostly expressed by liver cells, and while type 2 AIH autoantigens are specific to liver cells, type 1 AIH autoantigen are not [28]. Therefore, most models of autoimmune hepatitis that target specific autoantigens are models of type 2 AIH and have targeted either CYP2D6 or FTCD or both. These models have used adenoviral [29–31] or plasmid [32–36] vectors to express type 2 AIH liver autoantigen (CYP2D6 and/or FTCD) in mice. One of these models of type 2 AIH is based on xenoimmunization of wild type C57BL/6 mice with a plasmid coding for a chimeric human CYP2D6 and FTCD gene [36]. Using these human autoantigens, immune tolerance against the murine equivalent hepatic antigens could be broken and an autoimmune hepatitis triggered in naive, nontransgenic mice [36]. This model shows key characteristics of type 2 AIH in humans such as elevated levels of alanine aminotransferase (ALT), periportal, portal, and intralobular liver inflammatory infiltrates composed of mainly CD4^+^, but also CD8^+^ and B lymphocytes and anti-LKM1 and anti-LC1 antibodies ([36] and reviewed in [37]). Interestingly, as in type 2 AIH patients [38], ALT levels correlate with titers of anti-LC1 autoantibodies in this model [36].

In patients with AIH, as in those with multiple sclerosis [39] or lupus [40], low numbers of functional CD4^+^ Tregs have been reported [41]. Recently however, Peiseler et al. have reported that FOXP3^+^ Tregs in AIH are fully functional and not reduced in frequency [42]. These contradictory reports may have stemmed in part from the difficulty to distinguish between activated effector T cells and Tregs in humans based on markers like CD25 and FOXP3 which can be transiently expressed by activated effector T cells [43]. In addition, AIH treatment influences levels of Tregs as Treg frequency in adult AIH patients receiving immunosuppressive treatment was significantly reduced compared to both untreated AIH patients and healthy subjects [42]. Moreover, expression levels of CD25 seemed to be associated with disease activity in these AIH patients [42]. Recently, the group of Vergani described a decrease in frequency and functional impairments in CD39^+^ Treg subpopulations [44]. CD39^+^ Tregs show preferential suppression over CD4^+^ Th17 immunity and defective numbers of these cells have also been described in patients with multiple sclerosis [45]. These observations raise the question as to the role of these specialized CD39^+^ Tregs but also of Th17 effector cells in AIH pathogenesis. Further research is needed to confirm the impairments among Tregs in AIH patients and since disease activity and treatment can impact the number and phenotype of CD4^+^ Tregs, future studies should take great care in selecting and grouping their patients as well as using reliable and focused Treg markers.

**Figure 1:**
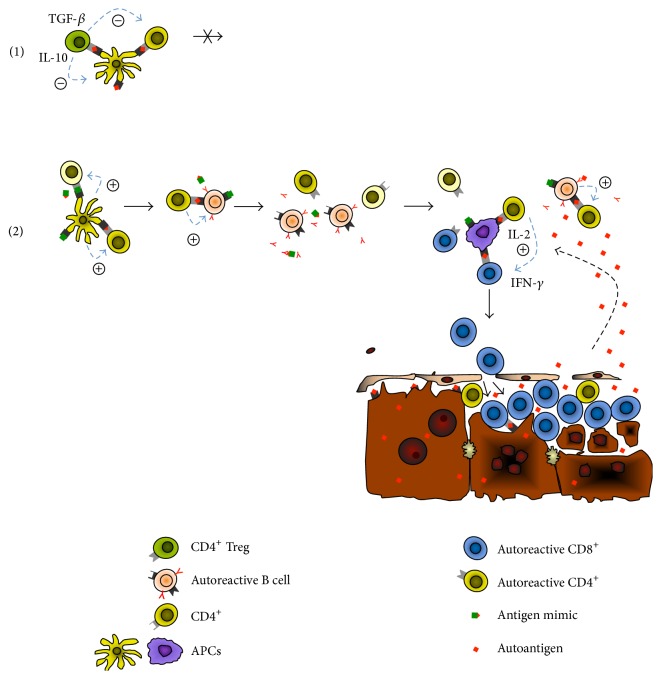
Role of CD4^+^ Tregs in the pathogenesis of autoimmune hepatitis. Based on data from experimental models and clinical observation we propose the following sequence of events leading to AIH through molecular mimicry. When an efficient peripheral tolerance to liver-expressed autoantigens (red) is induced either by preexisting natural Tregs or through the peripheral conversion of naïve CD4^+^ T cells to CD4^+^ Tregs (green), activation of autoreactive CD4^+^ T cells (yellow) would be abrogated and development of AIH prevented (1). However, when CD4^+^ Tregs are present in small numbers, activation through molecular mimicry of autoreactive CD4^+^ T cells against an antigen mimic (red/green) could occur leading to the activation of autoreactive B cells (pink) and production of autoantibodies (2). Autoreactive CD4^+^ T cells could then go on to induce an autoreactive CD8^+^ cytotoxic T cell response (blue) to liver-expressed autoantigens leading to hepatocyte lysis, release of autoantigens (red), and perpetuation of the T and B cell autoimmune response to the liver.

In mice, FOXP3 is a reliable marker for CD4^+^ Tregs [46]. In an experimental model of type 2 AIH, CD4^+^ Tregs have been found to influence the outcome of the disease [33, 34]. In this model of type 2 AIH, host factors were found to have a definite influence on liver injury encompassing both central and peripheral tolerance mechanisms [32–36]. Xenoimmunized C57BL/6 mice developed AIH but not 129 S/v mice after xenoimmunisation [33]. 129 S/v mice developed significantly higher numbers of Treg after xenoimmunization than C57BL/6 mice [33]. However, IL-10-secreting Tregs from 129 S/v and C57BL/6 mice were equally effective in controlling the proliferation of autoreactive CD8^+^ T cells [33]. These results suggest that the lack of peripheral tolerance against liver autoantigens in C57BL/6 mice does not result from a functional impairment but a lack of Tregs. Despite the presence of FTCD-specific autoreactive T cells, 129 S/v mice are able to prevent the development of AIH by controlling their proliferation* via *an increased Treg response [33]. Interestingly, xenoimmunized AIRE-invalidated mice did not develop AIH, despite reduced central tolerance against FTCD and related increase in FTCD-specific autoreactive T cells, owing to an increased number of Tregs [34]. Therefore, based on these observations, in opposition with thymic central tolerance, peripheral tolerance against liver autoantigens is paramount to disease susceptibility (Figure 1).

Remarkable efforts have been made to develop adoptive transfer methods of* ex vivo* expanded Tregs as a treatment for patients with autoimmune diseases [4]. In AIH, while not unanimous, many studies suggest that CD4^+^ regulatory T cells are present in fewer numbers and/or are functionally impaired in AIH patients [41, 47, 48]. In addition, functional human Tregs can be expanded* ex vivo *[49, 50]. Therefore, the use of* ex vivo* expanded Tregs to treat AIH patients has generated great enthusiasm [51]. However, to maximize the effectiveness and minimize unwanted side-effects, Tregs should be preferentially recruited by the inflamed liver and not diffused systemically [51]. Further research is needed on the status of regulatory T cells in patients with AIH. While animal models of AIH have benefited from regulatory T cells infusion [33], research is needed to assess the functionality of CD4^+^ regulatory T cells in patients with AIH and the link between disease activity and regulatory T cell levels. In addition, the development of AIH in humans may not only stem from lacking/dysfunctional CD4^+^ regulatory T cells and could also result from a resistance of effector cells to immune regulation [52]. These factors will need to be considered if Treg infusion is to be attempted in AIH patients.

CXCR3 mediates recruitment of Tregs to the liver through the expression of CXCL9 and CXCL10 by liver sinusoidal endothelium cells within a proinflammatory microenvironment, such as the one observed in the liver of patients with chronic hepatitis [53]. Recent studies have linked levels of circulating CXCL10 with disease activity in patients with AIH [54]. In mice, the lack of CXCR3 signalling leads to reduced recruitment of Tregs to the liver and an exacerbated liver disease [55]. Treg recruitment through the CXCR3 pathway is functional in AIH patients [53]. In mice with type 2 AIH, lack of Tregs in the liver of xenoimmunized C57BL/6 mice was not the result of a deficiency in their recruitment as CXCL9 and CXCL10 were abundantly expressed in the liver of these animals and their cognate receptor, CXCR3, was expressed by CD4^+^ Tregs [33]. Therefore, this model could be used to test the effectiveness of autologous* ex vivo* expanded CXCR3^+^ CD4^+^ Tregs infusion to treat AIH.

Tregs from AIH mice can be expanded* ex vivo* and maintain their functionality and CXCR3 expression [33]. Following infusion,* ex vivo* expanded CXCR3^+^ Tregs are rapidly recruited by the liver, restore peripheral tolerance, and significantly reduce liver inflammation [33]. One month after transfer, the numbers of Tregs in the liver of treated AIH mice were comparable to those of control mice suggesting that, upon the initial resolution of inflammation and restoration of immunological tolerance, high levels of Tregs were not necessary to maintain remission [33]. However, development of AIH in this model relies on xenoimmunization; therefore, further research is needed to assess whether the long-term maintenance of this immunological tolerance is conditional to the absence of a triggering factor (xenoimmunization) or if it could sustain repeated xenoimmunisation without developing severe AIH.

Based on these observations, infusion of autologous* ex vivo* expanded Tregs could be an effective therapeutic approach for the treatment of autoimmune hepatitis. Efforts are under way to expand antigen-specific Tregs for infusion in type 2 AIH patients [50, 56]. Since Treg recruitment through the CXCR3 pathway is functional in AIH patients [53], antigen-specific CXCR3^+^ Tregs could then target the inflamed liver hence potentiating the effectiveness of autologous Treg transfers. Alternatively,* in vivo* Treg expansion could be attempted using Trichostatin A treatment, a histone/protein deacetylases inhibitor able to expand and improve the suppressive function of regulatory T cells* in vivo* [57].

*Chronic Viral Hepatitis and Regulatory T Cells*. The liver is host to several chronic viral infections but infection does not always lead to viral persistence. Strong innate and adaptive immune responses can overcome viral escape mechanisms and, as in hepatitis A infection or resolved acute hepatitis B (HBV) or C virus (HCV) infections, achieve viral clearance. However, as observed in the majority of HCV infections (80%), viruses can evade these immune responses through high viral titers and inhibitory signalling leading to T cell exhaustion and viral persistence [58].

In HCV infections, the virus reaches its maximal titer several weeks before the induction of any detectable humoral or cellular immune responses and onset of liver disease [59]. In cases where HCV titers remain relatively low, T cell responses may remain undetectable even during chronic infection [59]. Recently, Park et al. have shown that HCV exposures at subinfectious levels in nonhuman primates could suppress T cell responses to subsequent acute infection challenge concomitant with quantitative and qualitative changes in regulatory T cells [60]. Thus, the liver's ability to induce tolerance to locally expressed antigens could contribute to the development of chronic liver infections by altering the immunological response to liver-expressed viral antigens.

Multiple factors contribute to reduced T cell responses found in chronically HCV-infected patients, notably, the induction and immune suppression by CD4^+^ Tregs [61]. Tregs expand during HCV infection in humans and this has been suggested to be involved in the establishment of a persistent infection [7, 62, 63]. CD4^+^CD25^+^ Tregs isolated from HCV-infected patients inhibit antiviral CD8^+^ T cell responses while depletion of CD4^+^ Tregs enhances proliferation of remaining T cells [64, 65]. Several studies have showed a correlation between increased CD4^+^ Treg numbers and chronic HCV infection [7, 64, 65]. However, in these studies, it remained unclear if these correlations stemmed from an HCV-specific tolerogenic response or from a nonspecific response induced by inflammation. The observation that exposure to HCV at subinfectious levels in nonhuman primates can lead to the induction of Tregs able to suppress HCV-specific effector T cells during subsequent HCV acute infection suggests that Tregs could directly impact viral clearance [60]. However, the small number of animals in this study did not allow a statistical comparison of the outcome of HCV challenge in preexposed and control animals [60].

**Figure 2:**
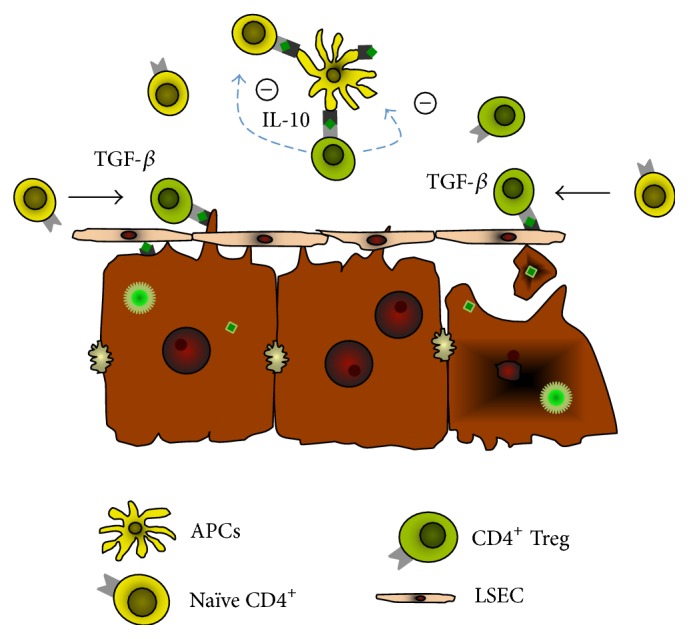
Role of CD4^+^ Tregs in chronic viral hepatitis. Based on data from chronically HCV-infected patients and experimental models, we propose the following role for CD4^+^ Tregs in chronic viral hepatitis. When liver cells are exposed to viral particles within the context of minimal innate immune responses and inflammatory signals, antigen presentation of virus-derived peptides by hepatocytes could occur with minimal costimulation and within the tolerogenic environment of the hepatic sinusoid (IL-10 and TGF-*β*) leading to the conversion of virus-specific naïve CD4^+^ T cells into Tregs (left). Antigen presentation by liver sinusoidal endothelial cells (LSEC) could also occur through the release of viral antigens by hepatocyte turnover leading to the conversion of virus-specific naïve CD4^+^ T cells into CD4^+^ Tregs. These virus-specific CD4^+^ Tregs could then inhibit the activation of virus-specific effector T cells and efficient antigen presentation by APCs through IL-10 secretion.

Antigen expression by the liver in absence of significant inflammation can lead to the conversion of CD4^+^ effector T cells to CD4^+^ regulatory T cells in a TGF-*β*-dependant fashion [8–10]. Therefore, expression by liver cells of HCV proteins in the early phase of infection could lead to a conversion of HCV-specific effector T cells into Tregs and establish a proper environment for viral persistence (Figure 2).

Studying the interactions of HCV and HBV with the immune system is complicated by the fact that these viruses do not naturally infect small mammals hence limiting their study to human subjects and nonhuman primates. However, other viruses that share several characteristics with HCV have been used to study the development of viral chronicity. Certain strains of lymphocytic choriomeningitis virus (LCMV; Armstrong, WE) lead to acute infections while others (LCMV strain docile and clone 13) can lead to chronic infections in wild type mice [66]. These differences between LCMV strains have been used extensively to study factors responsible for viral persistence. Studies using LCMV clone 13 allowed the description of T cell exhaustion as a viral escape mechanism [67], a mechanism subsequently described in human HBV, HCV, and HIV infections [68]. In a murine model of chronic infection with LCMV clone 13, CD4^+^ Treg depletion and PD-L1 blockade significantly reduced LCMV viral titers suggesting that Tregs could be involved in the persistence of LCMV clone 13 in mice [69].

To assess the ability of the liver to induce tolerance to a liver-expressed viral antigen and how it would impact the development of a chronic liver infection, a model of chronic viral hepatitis was developed using TTR-NP mice and LCMV infection [70]. TTR-NP mice express the nucleoprotein (NP) of LCMV specifically in hepatocytes and this leads to a strong peripheral tolerance against NP mediated by IL-10-secreting CD4^+^ Tregs. Despite an active immune response following infection with acute strains of LCMV (WE and Armstrong), Treg-mediated liver-induced peripheral tolerance against a single viral protein was sufficient to induce T cell exhaustion and chronic LCMV infection by limiting the antiviral T cell response in an otherwise immunocompetent host. Anti-CD25-mediated Treg depletion of chronically infected TTR-NP mice led to functional restoration of LCMV-specific CD4^+^ and CD8^+^ T cell responses and viral clearance.

This observation suggests that the liver can induce Treg-mediated peripheral tolerance to a locally expressed antigen, sufficiently impeding an antiviral immune response to lead to viral persistence and that Treg depletion in chronically infected animals is able to restore functionality of exhausted T cells.

While Tregs can impair the antiviral T cell response, they are also present to moderate T cell responses that could cause extensive tissue damage [71]. Therefore, an equilibrium must exist between viral clearance and protection from HCV-induced immunopathology in the response to HCV infection [61]. While elevated Tregs level may be beneficial during the chronic phase of HCV to minimize tissue damage, its inhibition of effective T cell response may hamper clearance of the virus therefore aiding viral persistence. This is similar to the type I interferon paradox during chronic viral infections where interferon-*α*/*β* signaling can both limit early viral replication, through inhibition of viral transcription and translation and through degradation of viral nucleic acids while also suppressing immune responses later during infection through the induction of IL-10, PD-L1 and indoleamine (2,3)-dioxygenase expression, thereby minimizing tissue damage [72, 73].

CD25 expressing Tregs could be targeted in chronically infected patients. Daclizumab, an anti-CD25 humanized antibody, is approved for human use and currently used to treat acute graft rejection by targeting CD25 expressing effector T cells [74]. However, treatment with daclizumab has been shown to effectively deplete Tregs and improve immune responses after tumor-antigen vaccination in patients with metastatic breast cancer [75]. Therefore, depletion of immunoregulatory T cells would be possible in chronically infected patients but further research is needed on the proper equilibrium needed between effective immune responses and minimization of virus-induced immunopathology in HCV-infected patients. Since regulatory T cells are important to maintain immunological tolerance to self-antigens, Treg depletion could affect peripheral tolerance to self-antigens. Careful monitoring of possible autoimmunity (antinuclear antibodies, cutaneous reactions) should be performed if daclizumab treatment is to be used in chronic HCV patients. Interestingly, it has recently been shown that daclizumab therapy maintains a limited population of regulatory T cells in humans and did not lead to adverse cutaneous events [76].

## 2. Conclusion

At the basis of both autoimmune diseases and chronic infections is an inappropriate immune response to an initial triggering factor. Overly tolerant immune responses can lead to chronic viral hepatitis [60, 70] while an overreactive and unbridled immune response can lead to autoimmune hepatitis [33, 44]. Therefore, it is a minor surprise that Tregs would be involved in both diseases' pathogenesis given that these cells are immune regulators and, thus, at the center of immune responses.

While further research is needed to ascertain the precise role of Tregs in the development of autoimmune hepatitis, these cells remain crucial in maintaining hepatic immunological tolerance and, as such, could prove to be of tremendous help to reestablish tolerance to liver-expressed autoantigens. Infusion of autologous* ex vivo* expanded CXCR3^+^ Tregs could be an effective therapeutic approach for the treatment of patients with autoimmune hepatitis. However, long-term maintenance of this tolerance would likely be conditional upon the absence of triggering factor(s). Therefore, further research is needed on elucidating the initiating triggers of AIH before long-term restoration of immunological tolerance in AIH patients can be considered.

While autoimmune responses are by definition inappropriate and thus would benefit from Treg immunosuppression, immune responses during chronic infections are a case where* timing is everything*. Regulatory mechanisms such as expression of inhibitory molecules (PD-1) by T cells and expansion of CD4^+^ Tregs can be necessary to prevent extensive tissue damage and immunopathology. However, if expression of viral antigens by liver cells can induce a state of peripheral tolerance mediated by Tregs contributing to the chronicity of the viral infection, therapeutic strategies targeting Tregs in patients chronically infected with hepatotropic viruses could represent a promising approach to restore functional antiviral immunity and clear infection. Further research is needed to assess the contribution of Tregs to the establishment and persistence of chronic HCV infection. Treg depletion at a proper time point could allow for effective antiviral immune responses, clearance of the virus, and restoration of a proper equilibrium between regulatory and effector immune responses.

## Abbreviations

HCV: Hepatitis C virus
HBV: Hepatitis B virus
LCMV: Lymphocytic choriomeningitis virus
Treg: Regulatory T cell
CTL: Cytotoxic T lymphocyte
ALT: Alanine aminotransferase.
